# Predictive Factors of Cytomegalovirus Colonic Reactivation in Patients with Active Ulcerative Colitis

**DOI:** 10.3390/v17040555

**Published:** 2025-04-11

**Authors:** Alexandre Jentzer, Aymeric Cantais, Xavier Roblin, Mathilde Barrau, Arnauld Garcin, Thomas Bourlet, Bruno Pozzetto, Sylvie Pillet

**Affiliations:** 1 CIRI—Centre International de Recherche en Infectiologie (GIMAP Team, University of Lyon, Univerity of Saint-Etienne, INSERM U1111, CNRS UMR5308, ENS de Lyon, UCBL1), Cedex 02, 42218 Saint-Etienne, France; alexandre.jentzer@gmail.com (A.J.); aymeric.cantais@chu-st-etienne.fr (A.C.); xavier.roblin@chu-st-etienne.fr (X.R.); thomas.bourlet@univ-st-etienne.fr (T.B.); sylvie.pillet@univ-st-etienne.fr (S.P.); 2 Department of Gastroenterology, University-Hospital of Saint-Etienne, Cedex 02, 42055 Saint-Etienne, France; mathilde.barrau@chu-st-etienne.fr; 3 Clinical Research, Innovation and Pharmacology Unit (URCIP), SNA/EPIS, Faculty of Medicine Jacques Lisfranc, Saint-Etienne University Hospital, Cedex 02, 42023 Saint-Etienne, France; arnauld.garcin@chu-st-etienne.fr; 4 Laboratory of Infectious Agents and Hygiene, University-Hospital of Saint-Etienne, Cedex 02, 42055 Saint-Etienne, France

**Keywords:** cytomegalovirus-associated colitis, ulcerative colitis, predictive factors, intestinal biopsy, CMV reactivation

## Abstract

Cytomegalovirus (CMV)-associated colitis reflects the adverse impact of CMV reactivation on ulcerative colitis (UC). Its diagnosis requires the detection of viral markers in intestinal biopsies sampled during endoscopy, which may constitute invasive and expensive analyses. Moreover, less than 30% of acute flare-ups in steroid refractory UC are associated with CMV colitis. This retrospective study aimed to identify non-invasive factors that are predictive of CMV reactivation, and was conducted from 2014 to 2019 in a cohort of UC patients consulting at the University Hospital of Saint-Etienne, France. Patient characteristics, disease activity, immunosuppressive treatment and tissue CMV DNA load were collected at the time of UC relapse. Factors potentially associated with CMV reactivation were analyzed through a multivariate analysis. A total of 173 UC patients providing 323 pairs of intestinal biopsies were analyzed. In the CMV seropositive subgroup, a Mayo endoscopic score ≥2 (OR 2.553, 95% CI 1.353–4.818, *p* = 0.004) was identified as a predictive factor of CMV colitis in the multivariate analysis; in contrast, biological parameters exhibited no predictive value. In addition, the use of anti-TNFα monoclonal antibodies was associated with a reduced risk of CMV reactivation (OR 0.384, 95% CI 0.158–0.935, *p* = 0.035). Intestinal biopsies appear to be unavoidable for assessing disease activity and CMV reactivation in UC patients.

## 1. Introduction

Cytomegalovirus (CMV) is a human opportunistic virus of the *Betaherpesvirinae* subfamily. It infects monocytes, endothelial cells, hematopoietic progenitors and other cells disseminated in tissues [1] that serve as viral reservoirs, especially in the latent state. Although the infection is most often contained by the immune system in immunocompetent individuals, CMV infection can severely impair the function of targeted organs such as the brain, retina, lung and digestive tract. CMV-associated disease is especially common in immunocompromised patients [2].

CMV reactivation in patients suffering from ulcerative colitis (UC) mainly results in CMV-associated colitis, which dramatically worsens the prognosis by “adding fuel to the fire” [3]. Indeed, CMV tissue reactivation can worsen the clinical course of the intestinal inflammatory disease, being associated with more severe disease, resistance to immunotherapy, and increased risks of hospitalization, colectomy and mortality in UC patients (reviewed in [4] and [5]). CMV-associated colitis reflects the particular adverse role of CMV reactivation in UC patients [4,6,7,8,9,10,11].

The diagnosis of CMV-associated colitis is accomplished through the detection of viral markers directly in the sampled intestine biopsies, as indicated in British [12] and European guidelines [13]: immunohistochemistry (IHC) or tissue quantitative PCR are currently recommended, with the molecular approach showing a higher sensitivity [4]. However, these analyses are invasive and expensive, and less than 30% of acute flare-ups of steroid refractory UC are linked with CMV colitis [6,14]. To reduce the need for endoscopy procedures and the associated costs, several studies have highlighted predictive factors of CMV colitis in UC patients. Patients with pancolitis have been identified as being at risk for CMV colitis [15,16,17]. A high Mayo endoscopic score is also a risk factor for CMV colitis [17,18,19,20,21], as well as the presence of mucosal ulcers [22]. Other studies have evaluated the roles of immunosuppressive therapies in CMV reactivation and, as expected, corticosteroid-based therapy was linked with an increased risk of CMV-associated colitis [15,17,19,20,22,23,24,25], whereas anti-tumor necrosis factor (TNF)-alpha administration was not [26,27,28]. It has also been found that UC patients with CMV colitis exhibit lower levels of hemoglobin [20,29] and albumin [17,29], as well as lower leucocyte [30] and eosinophil counts [17]. Altogether, the previous studies have revealed interesting elements that could allow for better identification of the risk of CMV-associated colitis in patients suffering from UC. Nevertheless, the studied populations were often heterogeneous, mixing patients with UC and Crohn’s disease (CD) [16,22,25,30,31,32]; however, CMV infection does not significantly impact the clinical course of CD [6,7,33]. Furthermore, disparate diagnostic techniques for CMV reactivation were used [15,16,17,30], including serological tests, IHC, and blood or tissue quantitative PCR, indicating their variable performance in terms of sensitivity and specificity [8].

The aim of this retrospective monocentric study was to evaluate clinical, endoscopic and biological factors in a cohort of UC patients with two intestinal biopsies in order to identify predictive factors of CMV reactivation.

## 2. Materials and Methods

### 2.1. Study Design and Patients

Consecutive patients consulting for moderate to severe UC, based on the European Crohn’s and Colitis Organization (ECCO) guidelines [13], at the Department of Gastroenterology of the University Hospital of Saint-Etienne between January 2014 and December 2019 were included in this retrospective study. All patients underwent a rectosigmoidoscopy, with sampling of 2 biopsies for CMV DNA load determination, as previously described [34].

All patient characteristics were recorded at the time of relapse, as defined by a gastroenterologist in the last medical report. The severity and extent of the disease were assessed according to patient records using the Mayo endoscopic score [21,35] and Montreal classification of the extent of UC [36], respectively. The Mayo endoscopic score includes 4 levels: 0, normal or inactive disease; 1, mild disease (erythema, decreased vascular pattern, mild friability); 2, moderate disease (marked erythema, absent vascular pattern, friability, erosions); and 3, severe disease (spontaneous bleeding, ulceration). When present, the number of endoscopic ulcerations was recorded. Immunosuppressive therapies (corticosteroids, 5-aminosalicylic acid, azathioprine, methotrexate, purine synthesis inhibitors, calcineurin, tacrolimus, anti-JAK [Janus kinase] and monoclonal therapies such as anti-TNFα, anti-integrin and ustekinumab) were also recorded. Steroid dependence was defined as an inability to reduce the dose of oral steroids below 10 mg/day. Steroid refractory UC was defined by the absence of clinical remission despite a full dose of corticosteroid therapy (0.75 mg/kg/day); in these patients, stopping steroids induced a flare-up. Biological parameters such as blood count, C-reactive protein (CRP) and liver markers were also analyzed.

### 2.2. CMV qPCR and CMV Serology

CMV DNA load was quantified via real-time quantitative polymerase chain reaction (qPCR) in 2 samples of inflamed tissue, as previously described [6,34]. CMV-associated colitis was defined by a viral load superior to 5 international units (IU)/100,000 cells in at least one biopsy of the sampled inflamed intestinal tissue [34]. Anti-CMV IgG antibodies were detected using an Architect i2000sr Immunoassay analyzer (Abbott); a patient was considered immune against CMV in the case of a positive antibody rate, according to the supplier’s threshold.

### 2.3. Statistical Analyses

The data were pre-processed and analyzed using Python libraries, including Pandas, NumPy 2.0.0 and StatsModels 0.15.0. The binary target variable was created by coding values greater than or equal to 5 as ’true’, and otherwise as ’false’. Following this, dummy variables were generated for categorical predictors, excluding the first category to avoid multicollinearity. Missing values were handled by excluding rows with any missing data. Logistic regression was applied to ascertain the impacts of various predictors on the likelihood of CMV colitis. A forward selection approach based on the Akaike Information Criterion (AIC) was utilized to identify the most informative predictors. The logistic regression model was fitted using the selected predictors with an added constant term for the intercept. Maximum likelihood estimation (MLE) was performed with an increased iteration limit in order to ensure convergence. The coefficients from the logistic regression model were exponentiated to obtain odds ratios (ORs), which express the change in odds of being ‘true’ holding all other variables constant. Confidence intervals (CIs) for these odds ratios were calculated and exponentiated as well, providing a range of values for the ORs. We estimated associated *p*-values for each predictor in the final model; *p*-values less than 0.05 were considered statistically significant.

## 3. Results

### 3.1. Patient Characteristics

**Figure 1:**
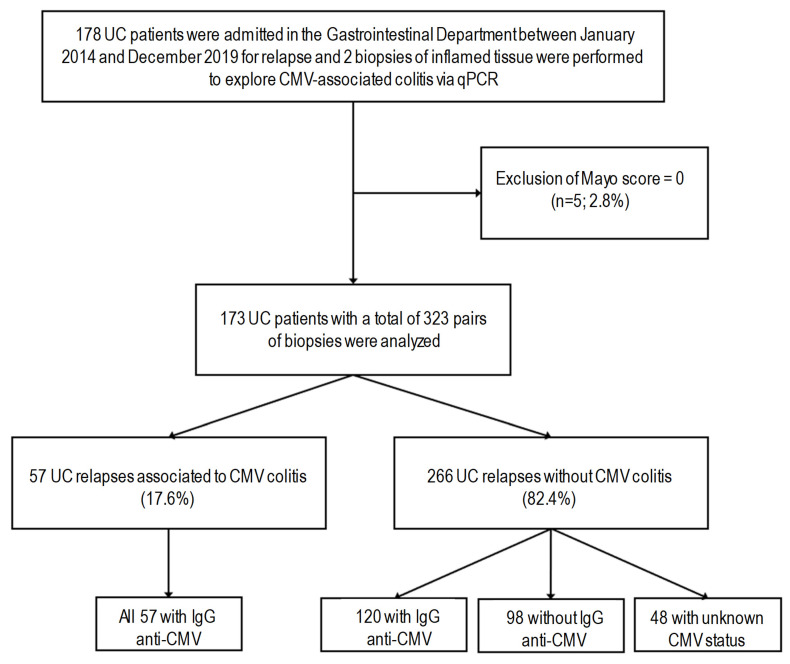
Flowchart of the study. The study was carried out on 173 UC patients, with a total of 323 pairs of biopsies. The predictive factors of CMV reactivation in multivariate analyses were assessed in the CMV seropositive subgroup, indicating 57 UC relapses associated with CMV colitis and 120 UC relapses without CMV colitis.

The study was carried out in 173 UC patients providing a total of 323 pairs of intestinal biopsies (Figure 1). Patients with detection of CMV DNA in the inflamed biopsy accounted for a total of 57 pairs of intestinal biopsies (17.6%); all presented a past CMV infection (IgG positive) at the time of biopsy. Of the 266 relapses without detection of CMV DNA in the biopsy (82.4%), 120 tested positive for anti-CMV IgG antibodies, 98 tested negative for this marker, and for 48, the CMV serological status was unknown (Figure 1).

The characteristics of relapses of UC, according to the CMV DNA load in biopsies from the CMV seropositive subgroup (potentially at risk of viral reactivation), are listed in Table 1. We observed severe disease activity, including a Mayo endoscopic score of 3 in 88 UC flare-ups and pancolitis in 82 UC flare-ups. Steroid dependence was found for 69 relapses and 24 relapses were steroid refractory. The most frequent treatments were anti-TNFα monoclonal antibodies, anti-integrin monoclonal antibodies and 5-aminosalicylic acid (5-ASA).

In the CMV seropositive subgroup (Table 1), as well as in the whole study population (Supplementary Table S1), we categorized the variables into 3 groups of clinical interest according to CMV viral load: a negative viral load: ≤5 IU/100,000 cells; a low positive viral load: [6–375] IU/100,000 cells; and a high positive viral load >375 IU/100,000 cells, according to the literature [6,34]. In the CMV seropositive subgroup, statistical differences were found between these groups only for the Mayo endoscopic score (*p* = 0.014), the presence of ulcers (*p* = 0.003), the measure of viral load (*p* < 0.001) and the lymphocyte count (*p* = 0.031).

### 3.2. Multivariate Analyses in the CMV Seropositive Subgroup

As detailed in Table 2, a Mayo endoscopic score ≥2 (OR 2.553, 95% CI 1.353–4.818, *p* = 0.004) was identified as a predictive factor of CMV colitis in the multivariate analysis. Unfortunately, the presence of ulcers was not a predictive factor of viral reactivation in our cohort, likely due to the lack of specificity in the relationship between CMV reactivation and ulcers. Pancolitis—which classifies UC at a severe state—was also not found as a predictive factor.

No biological parameter was identifiable as a potential biomarker of viral reactivation. Indeed, pathological variations in these parameters could be influenced by other diseases, making their interpretation complex.

Regarding therapies, neither steroid dependence nor steroid refractory UC were found to be predictive factors of CMV reactivation. In contrast, anti-TNFα monoclonal antibodies were associated with a reduction in CMV reactivation (OR 0.384, 95% CI 0.158–0.935, *p* = 0.035). A similar trend was observed for anti-integrin treatment, but without the statistical significance of the odds ratio (OR 0.359, 95% CI 0.111–1.156, *p* = 0.086); see Table 2.

## 4. Discussion

CMV colitis is diagnosed using specific markers (via IHC or tissue qPCR, as recommended [13]) directly in the sampled biopsies obtained through endoscopy, an invasive and cost-intensive analysis. Thus, the identification of risk factors for CMV reactivation could provide the means to reduce the need for biopsies.

In our study, none of the included patients exhibited primary CMV infection—a systemic infection that is mostly severe in adults and immunocompromised patients [8,37]. Our study included a homogeneous population with significant recruitment and CMV DNA load quantified via qPCR in biopsies, as recommended [13], which made it possible to have sufficient statistical power and avoid methodological bias. Several characteristics were studied in the subgroup of patients presenting positive rates of anti-CMV IgG, consequently being at risk of CMV colitis occurring during relapse.

The first characteristic described in the literature to be associated with CMV infection is age >30 years [22,30]. Both retrospective studies were performed in a limited number of patients with mixed UC and CD. We found that the age at biopsy sampling was not associated with the absence of CMV in the IgG anti-CMV positive subgroup. The effect of male sex was also inconsistent in the multivariate analysis.

Previous studies have shown that a severe active disease, represented by a high Mayo endoscopic score [16,19,20], the presence of ulcers [15,22,38] or pancolitis [15,16], is associated with CMV reactivation. Our study only identified the Mayo endoscopic score as a predictive factor of CMV reactivation in the multivariate analysis considering the CMV seropositive subgroup. Moreover, pancolitis—which classifies the pathology at a severe state—was not found to be a predictive factor of CMV colitis in our study. This pitfall could be explained by the retrospective character of the study; indeed, very severe diseases could have been missed as surgery was performed before the biopsies were sampled for CMV evaluation. Nevertheless, the Mayo endoscopic score, presence of ulcers and pancolitis are variables requiring endoscopy; therefore, clinicians cannot be informed in advance whether patients require a biopsy or not.

Regarding immunotherapies, steroid dependence and steroid refractory UC were not associated with viral colitis in our study, possibly due to the administration of a low dose of steroid at the time of the biopsy and early switching to one of the other immunotherapies, which are now available to treat UC flare-ups. Indeed, steroid-induced immunosuppression is known to lead to CMV reactivation only at high doses or during long-lasting treatment [15,17,19]. Notably, the use of anti-TNFα monoclonal antibodies was shown to be associated with a reduced risk of CMV reactivation in this study, which is coherent with the existing literature [15,17,22,27,31]. Indeed, TNFα is a molecule synthesized in large quantities in the colon of patients with UC, and this inflammatory cytokine is a major initiator of the transition from viral latency to a lytic phase. Anti-TNFα monoclonal antibodies therefore act by reducing inflammation and potentially slowing down the transition to the lytic phase.

In the literature, several blood markers—such as hemoglobin [20], albumin [15,17] and eosinophil count [17]—have been considered as predictive factors of CMV reactivation, although with contradictory results. In our retrospective cohort, biological markers were not shown to be informative regarding the risk of CMV reactivation.

This study had some limitations. In addition to the retrospective design and monocentric nature of the investigations, a limited number of biological parameters were explored, corresponding to those that had been recorded in all the patients’ files. For instance, aside from CRP or white blood cell counts, we acknowledge that more sophisticated inflammatory response markers would have been useful to analyze this key determinant of CMV reactivation in the course of UC. In the design of further studies, it will be necessary to obtain a series of additional biomarkers that could be important to record prospectively, with the aim of evaluating their prognostic value as a non-invasive proxy for the prediction of CMV reactivation in the context of UC.

## 5. Conclusions

Based on the presented results, it seems difficult to predict CMV reactivation during a UC relapse without requiring endoscopy to assess the activity of the disease and determine the presence of CMV in biopsies. We highly recommend performing CMV serology first in order to determine whether a previous CMV infection could reactivate and induce CMV-associated colitis, as IgG-negative patients do not have such risks (except in the case of primary infection). Future research may focus on the specific anti-CMV immune response, which has been poorly explored to date [39,40] but could help in classifying patients at high risk of viral colitis. Similarly, it has been shown that CMV can play a role in oncogenesis via oncomodulation [41,42]; as such, it could be interesting to investigate the role of this specific signaling pathway in the occurrence of flare-ups in the course of UC.

## Figures and Tables

**Table 1:** Characteristics of ulcerative colitis (UC) patients according to the CMV DNA load in biopsy for the CMV seropositive subgroup.

Variable	CMV DNA Load in Biopsy (IU/100,000 Cells)			*p*-Value
	≤5	6–375	>375	
Pair of biopsies: number	120	25	32	-
Gender: number of males (%)	51 (42.5)	11 (44)	9 (28.1)	0.308
Age at biopsy in years: mean (SD)	45.45 (18.49)	46.6 (19.35)	47.75 (15.35)	0.804
Duration of the disease at the biopsy: mean (SD)	37.58 (17.37)	38.8 (18.96)	41.16 (15.72)	0.579
Disease description Number of relapses per patient during the study *: number (%)				0.504
1	56 (46.7)	13 (52)	16 (50)	
2	29 (24.2)	3 (12)	11 (34.4)	
3	20 (16.7)	3 (12)	1 (3.1)	
>3	14 (12.4)	6 (24)	4 (12.5)	
Mayo endoscopic score: number (%)				0.014
1	24 (20)	2 (8)	2 (6.2)	
2	47 (39.2)	6 (24)	8 (25)	
3	49 (40.8)	17 (68)	22 (68.8)	
Presence of ulcers: number (%)	50 (41.7)	16 (64)	23 (71.9)	0.003
Montreal classification: number (%)				0.453
E1 (proctitis)	19 (15.8)	4 (16)	1 (3.1)	
E2 (left-sided colitis)	47 (39.2)	10 (40)	14 (43.8)	
E3 (pancolitis)	54 (45)	11 (44)	17 (53.1)	
Viral load expressed as IU/100,000 cells: mean (SD)	0.46 (1.18)	56.36 (68.57)	14,990.47 (31,785.28)	<0.001
Therapeutic used at the time of flare-up: number (%)				
Steroid dependence	45 (37.5)	9 (36)	15 (46.9)	0.594
Steroid refractory	16 (13.3)	3 (12)	5 (15.6)	0.917
5-ASA	21 (17.5)	7 (28)	5 (15.6)	0.419
Purine synthesis inhibitors	10 (8.3)	1 (4)	5 (15.6)	0.282
Anti-TNFα monoclonal therapy	54 (45)	7 (28)	13 (40.6)	0.289
Anti-integrin monoclonal therapy	23 (19.2)	6 (24)	2 (6.2)	0.152
Biological parameters: mean (SD)				
Hemoglobin (g/dL)	13.29 (2.08) (n = 87)	12.54 (1.67) (n = 20)	12.92 (2.1) (n = 26)	0.300
White blood cells (10^9^/L)	8.36 (2.98) (n = 87)	7.73 (1.87) (n = 20)	9.59 (2.1) (n = 25)	0.054
Lymphocyte (10^9^/L)	2.16 (0.7) (n = 88)	1.91 (0.62) (n = 20)	2.49 (0.91) (n = 25)	0.031
Neutrophils (10^9^/L)	5.3 (2.79) (n = 87)	4.86 (1.81) (n = 20)	3.1 (1.98) (n = 25)	0.232
Eosinophils (10^9^/L)	0.19 (0.2) (n = 80)	0.2 (0.21) (n = 19)	0.14 (0.22) (n = 24)	0.467
Platelets (10^9^/L)	322.88 (122.75) (n = 80)	343.47 (132.47) (n = 19)	313.46 (85.58) (n = 24)	0.700
CRP (mg/L)	15.11 (30.01) (n = 87)	17.99 (18.13) (n = 20)	17.52 (18.17) (n = 24)	0.889
ASAT (IU/L)	23.87 (11.9) (n = 86)	20 (9.98) (n = 20)	24.25 (10.79) (n = 24)	0.363
ALAT (IU/L)	22.26 (17.36) (n = 87)	22.4 (28.53) (n = 20)	26.4 (18.74) (n = 25)	0.643
Alkaline phosphatase (IU/L)	74.09 (27.62) (n = 86)	77.95 (28.85) (n = 20)	75.26 (46.54) (n = 23)	0.887

ALAT: alanine aminotransferase; ASA: 5-Aminosalicylic acid; ASAT: aspartate aminotransferase; IU: international units; SD: standard deviation; TNFα: tumor necrosis factor alpha. * A same patient can present several relapses in the course of the study, with CMV status potentially differing between two relapses.

**Table 2:** Predictive factors of CMV reactivation in multivariate analysis in the CMV seropositive subgroup.

Variable	Odds Ratio	95% Confidence Interval	*p*-Value
Mayo endoscopic score ≥ 2	2.553	1.353–4.818	0.004
Anti-TNFα monoclonal antibodies	0.384	0.158–0.935	0.035
Anti-integrin monoclonal antibodies	0.359	0.111–1.156	0.086

This table represents the studied variables for predictive factors of CMV reactivation in the multivariate analysis considering the CMV seropositive subgroup. Odds Ratio with confidence interval (95% CI) and *p*-value are shown. An OR < 1 represents a protective factor and an OR > 1 represents a risk factor of viral colitis. TNFα: tumor necrosis factor alpha.

## Data Availability

The data presented in this study are available on request from the corresponding author due to privacy restrictions.

## Supplementary Materials

The following supporting information can be downloaded at https://www.mdpi.com/article/10.3390/v17040555/s1: Table S1: Characteristics of ulcerative colitis (UC) patients according to the CMV DNA load in biopsy in the whole study population.
