# Supplementary material for: Predictive Factors of Cytomegalovirus Colonic Reactivation in Patients with Active Ulcerative Colitis
Source: Viruses. 2025 Apr 11;17(4):555. doi: 10.3390/v17040555 (PMC12031004; doi:10.3390/v17040555)
Supplement: Supplementary file 1 [file viruses-17-00555-s001.zip › viruses-3549755-supplementary.pdf]

**Table S1.** Characteristics of ulcerative colitis (UC) patients according to the CMV DNA load in biopsy in the whole study population.

| Characteristics | CMV DNA load in biopsy (IU/100,000 cells) | | | p-value |
|---|---|---|---|---|
| | ≤ 5 | 6 - 375 | > 375 | |
| **Pairs of biopsies: number** | 266 | 25 | 32 | - |
| **Gender: number of males (%)** | 117 (44.3) | 13 (48.1) | 9 (28.1) | 0.186 |
| **Age at biopsy in years: mean (SD)** | 36.22 (16.5) | 34.26 (19.04) | 41 (15.72) | 0.283 |
| **Duration of the disease at the biopsy: mean (SD)** | 44.35 (17.11) | 45.22 (19.24) | 48 (15.35) | 0.564 |
| **Disease description** | | | | |
| Number (%) of relapses per patient during the study*: number (%) | | | | |
| 1 | 141 (53.4) | 14 (51.9) | 16 (50) | |
| 2 | 60 (22.7) | 3 (11.1) | 11 (34.4) | 0.346 |
| 3 | 34 (12.9) | 3 (11.1) | 1 (3.1) | |
| >3 | 29 (10.9) | 7 (25.9) | 4 (12.4) | |
| Mayo endoscopic score: number (%) | | | | **0.029** |
| 1 | 38 (14.4) | 2 (7.4) | 2 (6.2) | |
| 2 | 109 (41.3) | 7 (25.9) | 8 (25) | |
| 3 | 117 (44.3) | 18 (66.7) | 22 (68.8) | |
| Presence of ulcerations: number (%) | 114 (43.2) | 17 (63) | 23 (71.9) | **0.002** |
| Montreal classification: number (%) | | | | 0.626 |
| E1 (proctitis) | 30 (11.4) | 4 (14.8) | 1 (3.1) | |
| E2 (left-sided colitis) | 109 (41.4) | 10 (37) | 14 (43.8) | |
| E3 (pancolitis) | 124 (47.1) | 13 (48.1) | 17 (53.1) | |
| Viral load expressed as IU/100,000 cells: mean (SD) | 0.22 (0.86) | 55.7 (66.43) | 14990.47 (31785.28) | **<0.001** |
| **Therapeutic status at the time of flare-up: number (%)** | | | | |
| Steroid dependency | 80 (30.3) | 11 (40.7) | 15 (46.9) | 0.337 |
| Steroid refractory status | 52 (19.7) | 3 (11.1) | 5 (15.6) | 0.800 |
| 5-aminosalicylic acid (5-ASA) therapy | 56 (21.2) | 7 (25.9) | 5 (15.6) | 0.620 |
| Purine synthesis inhibitor therapy | 22 (8.3) | 1 (3.7) | 5 (15.6) | 0.242 |
| Anti-TNF$\alpha$ mAb therapy | 101 (38.3) | 8 (29.6) | 13 (40.6) | 0.638 |
| Anti-integrin mAb therapy | 44 (16.7) | 7 (25.9) | 2 (6.2) | 0.122 |
| **Biological parameters: mean (SD)** | | | | |
| Hemoglobin (g/dL) | 13.16 (1.97) (n=189) | 12.55 (1.59) (n=21) | 12.92 (2.1) (n=25) | 0.347 |
| White blood cells ($10^9$/L) | 8.65 (2.98) (n=188) | 7.96 (1.97) (n=22) | 9.59 (2.1) (n=24) | 0.136 |
| Lymphocytes ($10^9$/L) | 1.92 (0.81) (n=189) | 2.11 (0.87) (n=21) | 2.49 (0.91) (n=24) | **0.005** |
| Neutrophils ($10^9$/L) | 5.81 (2.99) (n=188) | 4.81 (1.72) (n=22) | 6.1 (1.98) (n=24) | 0.235 |
| Eosinophils ($10^9$/L) | 0.2 (0.23) (n=179) | 0.27 (0.35) (n=22) | 0.14 (0.22) (n=23) | 0.187 |
| Platelets ($10^9$/L) | 336.45 (147.13) n=179) | 348.29 (126.76) (n=22) | 313.46 (85.58) (n=24) | 0.678 |
| CRP (mg/L) | 17.46 (33.92) (n=185) | 16.63 (17.80) (n=22) | 17.52 (18.17) (n=24) | 0.991 |
| ASAT (IU/L) | 23.49 (11.49) (n=183) | 19.73 (9.58) (n=22) | 24.25 (10.79) (n=23) | 0.300 |
| ALAT (IU/L) | 25.4 (23.11) (n=183) | 21.64 (27.25) (n=22) | 26.40 (18.74) (n=24) | 0.740 |
| Alkaline phosphatase (IU/L) | 74.2 (29.46) (n=181) | 84.23 (34.54) (n=22) | 75.26 (46.54) (n=22) | 0.382 |

ALAT: alanine aminotransferase; ASAT: aspartate aminotransferase; CRP: C-reactive protein; IU: international units; SD: standard deviation; mAb: monoclonal antibody; TNF$\alpha$: tumor necrosis factor alpha.

*A same patient can present several relapses in the course of the study, with CMV status potentially differing between two relapses.
